# Fluctuation in radioresponse of HeLa cells during the cell cycle evaluated based on micronucleus frequency

**DOI:** 10.1038/s41598-020-77969-0

**Published:** 2020-11-30

**Authors:** Hiroaki Shimono, Atsushi Kaida, Hisao Homma, Hitomi Nojima, Yusuke Onozato, Hiroyuki Harada, Masahiko Miura

**Affiliations:** 1 Division of Oral Health Sciences, Department of Oral Radiation Oncology, Graduate School of Medical and Dental Sciences, Tokyo Medical and Dental University, 1-5-45 Yushima, Bunkyo-ku, Tokyo, 113-8549 Japan; 2 Division of Oral Health Sciences, Department of Oral and Maxillofacial Surgery, Graduate School of Medical and Dental Sciences, Tokyo Medical and Dental University, 1-5-45 Yushima, Bunkyo-ku, Tokyo, 113-8549 Japan

**Keywords:** Cancer, Cell biology

## Abstract

In this study, we examined the fluctuation in radioresponse of HeLa cells during the cell cycle. For this purpose, we used HeLa cells expressing two types of fluorescent ubiquitination-based cell cycle indicators (Fucci), HeLa-Fucci (CA)2 and HeLa-Fucci (SA), and combined this approach with the micronucleus (MN) assay to assess radioresponse. The Fucci system distinguishes cell cycle phases based on the colour of fluorescence and cell morphology under live conditions. Time-lapse imaging allowed us to further identify sub-positions within the G1 and S phases at the time of irradiation by two independent means, and to quantitate the number of MNs by following each cell through M phase until the next G1 phase. Notably, we found that radioresponse was low in late G1 phase, but rapidly increased in early S phase. It then decreased until late S phase and increased in G2 phase. For the first time, we demonstrated the unique fluctuation of radioresponse by the MN assay during the cell cycle in HeLa cells. We discuss the difference between previous clonogenic experiments using M phase-synchronised cell populations and ours, as well as the clinical implications of the present findings.

## Introduction

It has been about 60 years since Terasima and Tolmach reported that radiosensitivity fluctuates during the cell cycle in proliferating HeLa cells in vitro^1–3^. In those studies, the most radioresistant phases were early G1, late S, and G2, and the most sensitive phases were the G1/S boundary and M phase. Subsequently, Sinclair et al. reported that the most radioresistant phase was late S and the most sensitive phases were G2 and M; these experiments mainly used Chinese hamster cells (V79) with very short G1 phases^4–7^. Interestingly, sensitive phases around the G1/S boundary were commonly observed only in cells with a longer G1 phase, like HeLa^7^. Most such studies were performed using cell populations synchronised in M phase by the shake-off method, which is based on cells detaching from the culture dish due to the round shape of mitotic cells^1–7^. This method does not induce any significant stresses, including DNA damage, but is still limited by technical issues related to the yield of M-phase cells and subsequent sustainability of synchronicity. Sinclair et al. reported that additional procedures are required to keep purity high^4,5^. That said, even if a highly pure M phase-synchronised cell population is obtained, the purity of the cell population will subsequently decrease, causing collapse of synchronicity and diminishing experimental precision. Therefore, it is necessary to evaluate much purer cell populations representing specific cell cycle phases that have not been exposed to any significant stresses.

The fluorescent ubiquitination-based cell cycle indicator (Fucci) is an innovative method for visualising the cell cycle under live conditions. The method takes advantage of cell cycle-dependent ubiquitination of Cdt1 and Geminin by the E3 ligases of SCF^Skp2^ and APC^Cdh1^, respectively^8^. In this system, cells in G1 and S/G2/M phases emit red [from monomeric Kusabira Orange 2 (mKO2)] and green [from monomeric Azami Green (mAG)] fluorescence, respectively. In addition, cells in early S phase can be distinguished because they emit both types of fluorescence. Although this method cannot distinguish S and G2 phases, we previously showed detailed radiation-induced kinetics of accumulation of cells in green phase using HeLa-Fucci (SA) cells and that it was mainly attributed to G2 arrest^9,10^. HeLa cells do not undergo G1 arrest due to loss of p53 function caused by human papilloma virus infection, allowing easy visualisation of G2 arrest kinetics. The combination of Fucci with time-lapse imaging allowed us to determine sub-positions of the cell cycle during G1 phase at the time of irradiation by measuring the time between irradiation during red phase and the start of the green phase^10^. Recently, the next generation of the Fucci system was developed, designated Fucci (CA)2, which uses the E3 ligase of CUL4^Ddb1^ instead of SCF^Skp2^. Consequently, S and G2 phases are distinguishable, as cells in G2/M phases emit both red (from mCherry) and green (from mVenus) fluorescence^11^. We anticipated that with this new system, the same strategy used for G1 phase could also be applied to cells in S and G2 phases at the time of irradiation.

A micronucleus (MN) is a small nucleus-like structure formed when cells carrying DNA damage, such as double-strand breaks (DSBs), divide during mitosis^12^. MN frequency is a useful marker for genomic integrity, and many studies have shown that it is strongly correlated with radiosensitivity^12–14^. Radiosensitivity is usually determined by colony assays and surviving fractions determined by such assays are strongly associated with the DSB repair activities of the non-homologous end joining and homologous recombination pathways^15^. Because the number of residual DSBs is thought to determine MN frequency^15^, it is reasonable that MN frequency reflects radiosensitivity. Furthermore, the number of MNs can be quantitated by tracing each cell in mitosis and subsequent G1 phase. Although the MN assay is not equivalent to a colony assay, because it cannot evaluate clonogenic potential, these considerations raise the possibility that combining the Fucci system and the MN assay would provide additional insight into the precise fluctuation of radiosensitivity during the cell cycle.

In this study, we succeeded in determining the radioresponse from G1 to G2 phase by taking advantage of the properties of Fucci. We observed similar decreases of radioresponse from the early to late stages of both G1 and S phases, accompanied by a rapid change from late G1 to early S phase. We discuss the difference between previously reported clonogenic results and ours, as well as the clinical implications of the present findings.

## Results

### Validation of sustainability of synchronicity in HeLa-Fucci cells virtually synchronised in M phase

**Figure 1:**
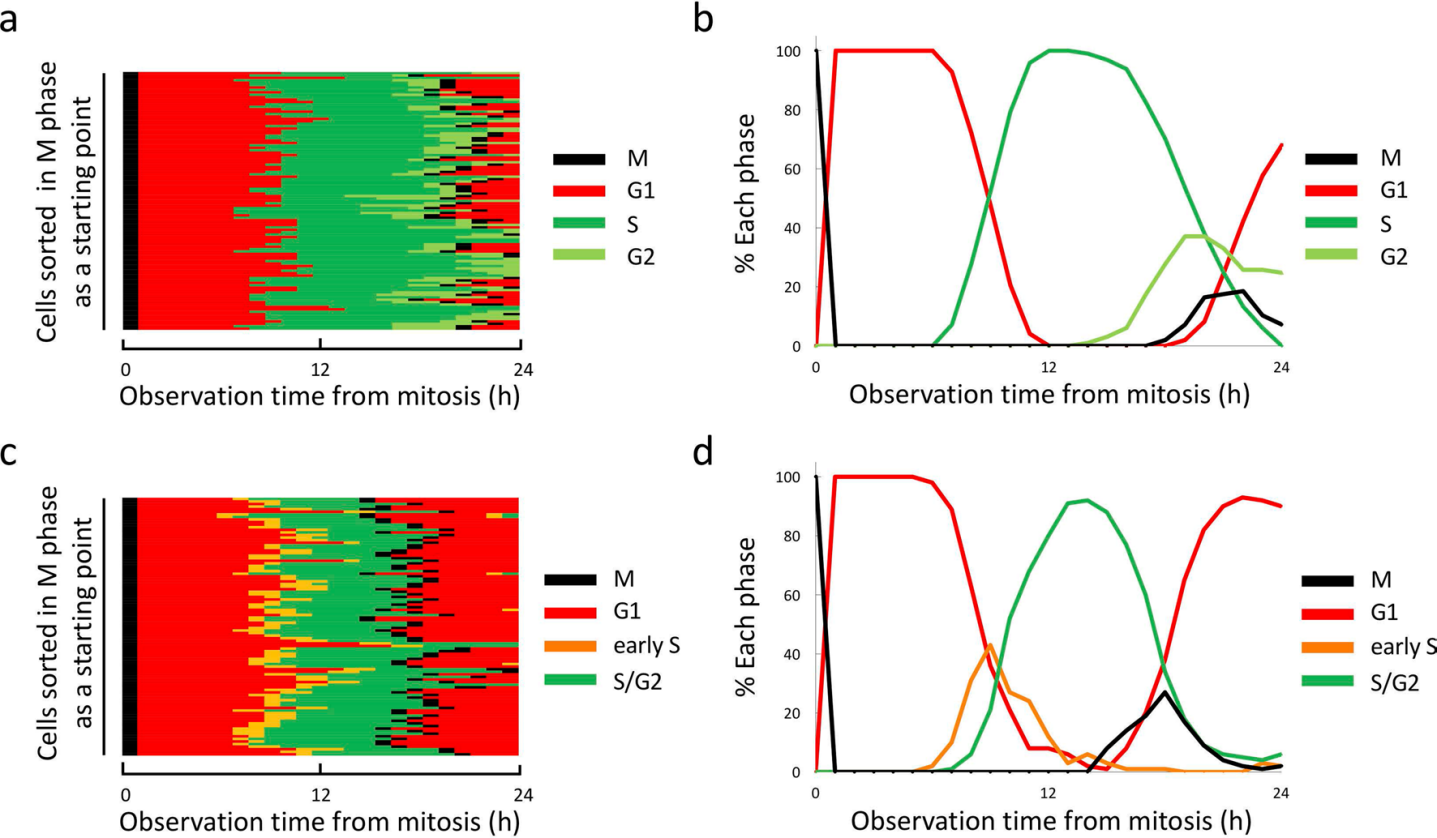
Sustainability of synchronicity of virtually sorted cells in M phase for HeLa-Fucci (CA)2 and HeLa-Fucci (SA) cells. (**a**) Pedigrees for HeLa-Fucci (CA)2 cells sorted in M phase as a starting point. From time-lapse imaging data, 107 cells in M phase were selected, and cell cycle changes thereafter were depicted. Each cell was sorted so as to start in M phase. (**b**) Time course of cell cycle synchronicity. Proportions of cells in each cell cycle phase were plotted against time after M phase. (**c**) Pedigrees for HeLa-Fucci (SA) cells sorted in M phase as a starting point. From time-lapse imaging data, 100 cells in M phase were selected, and cell cycle changes thereafter were depicted. Each cell was sorted so as to start in M phase. (**d**) Time course of cell cycle synchronicity. Proportions of cells in each cell-cycle phase were plotted against time after M phase.

The loose attachment of cells to dishes during mitosis allows M-phase cells to be selectively detected by the shake-off method^1–3^, with quite a high purity (> 90%), which has been used in a wide range of studies^16,17^. However, sustainability of the synchronicity was not fully investigated. Data derived from time-lapse imaging allowed us to virtually reconstitute a completely M phase-synchronised cell population, and it was possible to follow the collapse of synchronicity over time. Figure 1a represents pedigrees for each cell that was synchronised in M phase at time 0 in HeLa-Fucci (Ca)2 cells. All cells entered G1 phase at the same time and were in G1 phase for up to 6 h, but then some cells began to progress into S phase. Notably, 9 h after the start of observation, the ratio of G1 to S phase cells was about 1:1. From 12–14 h, S phase cells accounted for approximately 100% of cells, but thereafter, G2-phase cells gradually increased and reached a proportion of 40% by around 20 h (Fig. 1b). These findings clearly demonstrate that synchronicity is easily collapsed, even if we begin with a perfectly synchronised cell population. In particular, at the G1/S and S/G2 boundaries, the population consists of equally mixed phases of cells. Essentially the same findings were obtained from HeLa-Fucci (SA) cells (Fig. 1c,d). Given that the fluctuation of radiosensitivity was originally determined using a cell population synchronised in M phase by the shake-off method, previously published findings must be re-evaluated using much purer cell populations.

### Characterisation of G1, S, and G2 arrests in HeLa-Fucci (CA)2 cells irradiated in G1, S, or G2 phase

**Figure 2:**
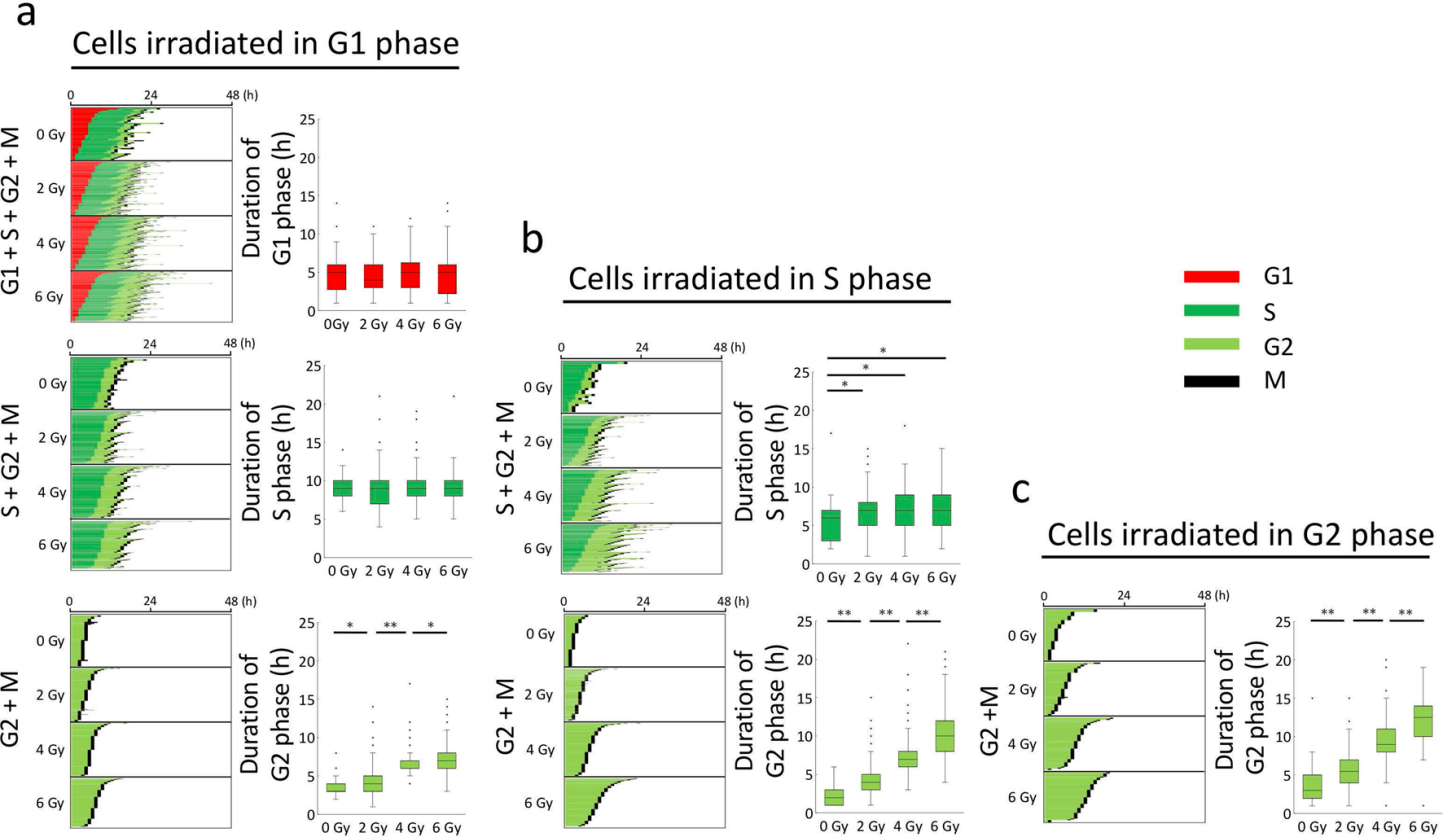
Characteristics of cell cycle arrests in G1, S, and G2 phases in cells irradiated in G1, S, and G2 phases. Pedigrees were made from the start of observation until the next M phase using time-lapse imaging data for cells unirradiated and irradiated in G1 (**a**), S (**b**), and G2 (**c**) phases following the indicated doses. Cells were then sorted according to the lengths of G1, S, and G2 phases (left panels). Each duration is represented as a box-whisker plot showing outliers, distribution intervals, 25–75% interquartile range (box), and median (right panels). Mann–Whitney U-test: *p < 0.05; **p < 0.01. Cell numbers for each radiation dose group: (**a**) 50–232; (**b**) 25–218; (**c**) 25–61. The smallest cell number was for 0 Gy.

We previously reported that the durations of the red phase do not change even when HeLa-Fucci (SA) cells are irradiated with up to 10 Gy^10^. This indicates that G1 arrest never occurs, and that cells irradiated in G1 phase enter S phase normally, presumably carrying DNA damage. Pedigrees were constructed from time-lapse imaging data of the whole cell population of HeLa-Fucci (CA)2 cells following various doses of irradiation, and the cells were subsequently sorted according to the lengths of their G1, S, and G2 phases. In Fig. 2a, cells were irradiated in G1 phase, and the duration of G1 phase did not change at all up to a dose of 6 Gy (upper panels); however, cells subsequently entering S phase (middle panels) were not affected, and the proportion of cells entering G2 phase (lower panels) was clearly related to dose. Cells irradiated in S phase (Fig. 2b, upper and lower panels) showed significantly higher durations of both S and G2 phases, but only the latter increase was dose-dependent. Cells irradiated in G2 phase (Fig. 2c) also increased the duration of G2 phase in a dose-dependent manner. Thus, our previous findings were confirmed: in HeLa-Fucci (CA)2 cells, G2 arrest was most prominent among the three typical cell-cycle arrests.

### Detection and quantitation of micronucleus using the Fucci system

**Figure 3:**
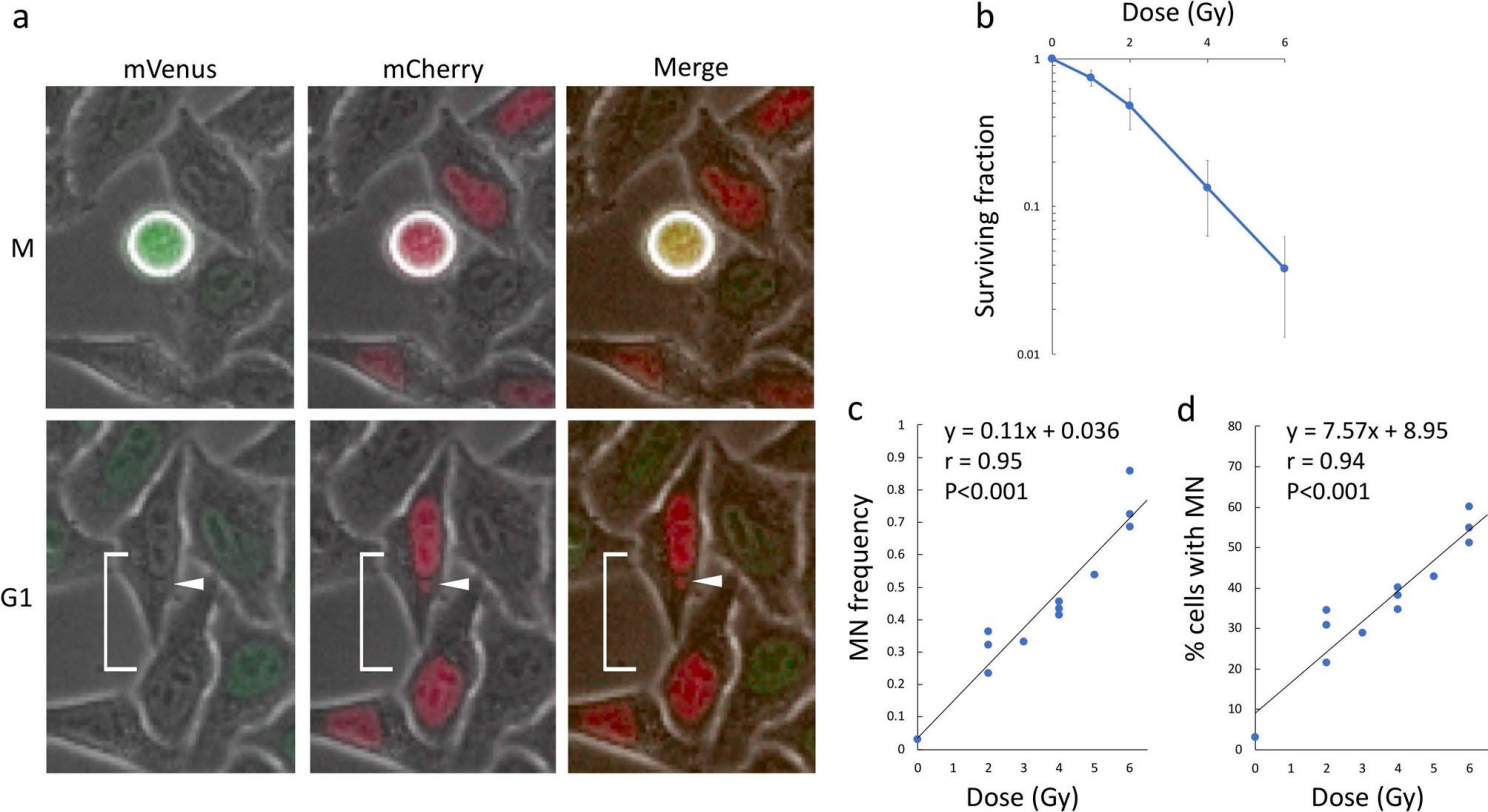
Detection of MN and its dose response. (**a**) Detection of MN in HeLa-Fucci (CA)2 cells following irradiation. Radiation (2 Gy)-induced MN, formed in G1 phase via M phase, is shown on the time-lapse images. Arrowhead, MN. white bracket, paired-daughter cells. (**b**) Dose–survival curve in HeLa-Fucci (CA)2 cells. Surviving fractions were determined by colony formation assay. Data are means ± SD of three independent experiments. (**c**) Relationship between radiation dose and MN frequency (mean MN number per two daughter cells after mitosis). (**d**) Relationship between radiation dose and proportions of cells with MNs (percentages of paired-daughter cells that contained MNs in all counted paired-ones).

To determine radioresponse, we performed micronucleus (MN) assays, as MNs can be counted in time-lapse images. Indeed, it was possible to clearly identify MNs immediately after mitosis (Fig. 3a) after 2 Gy of irradiation. Cytochalasin B is usually used to form binucleated cells to distinguish mitosis-associated formation of MNs^12^; however, this was unnecessary in this study because time-lapse imaging enabled us to follow cells through the transition from mitosis to G1 phase. A dose-survival curve is shown in Fig. 3b, revealing a typical pattern consisting of a curve with a shoulder. The relationship between radiation dose and MN frequency was linear, as also reported by other studies^13^ (Fig. 3c). Similarly, radiation dose and proportions of cells with MNs were highly correlated (Fig. 3d).

### Determination of sub-positions within G1 and S phases at the time of irradiation, according to the time until the next cell cycle phase and corresponding MN levels

**Figure 4:**
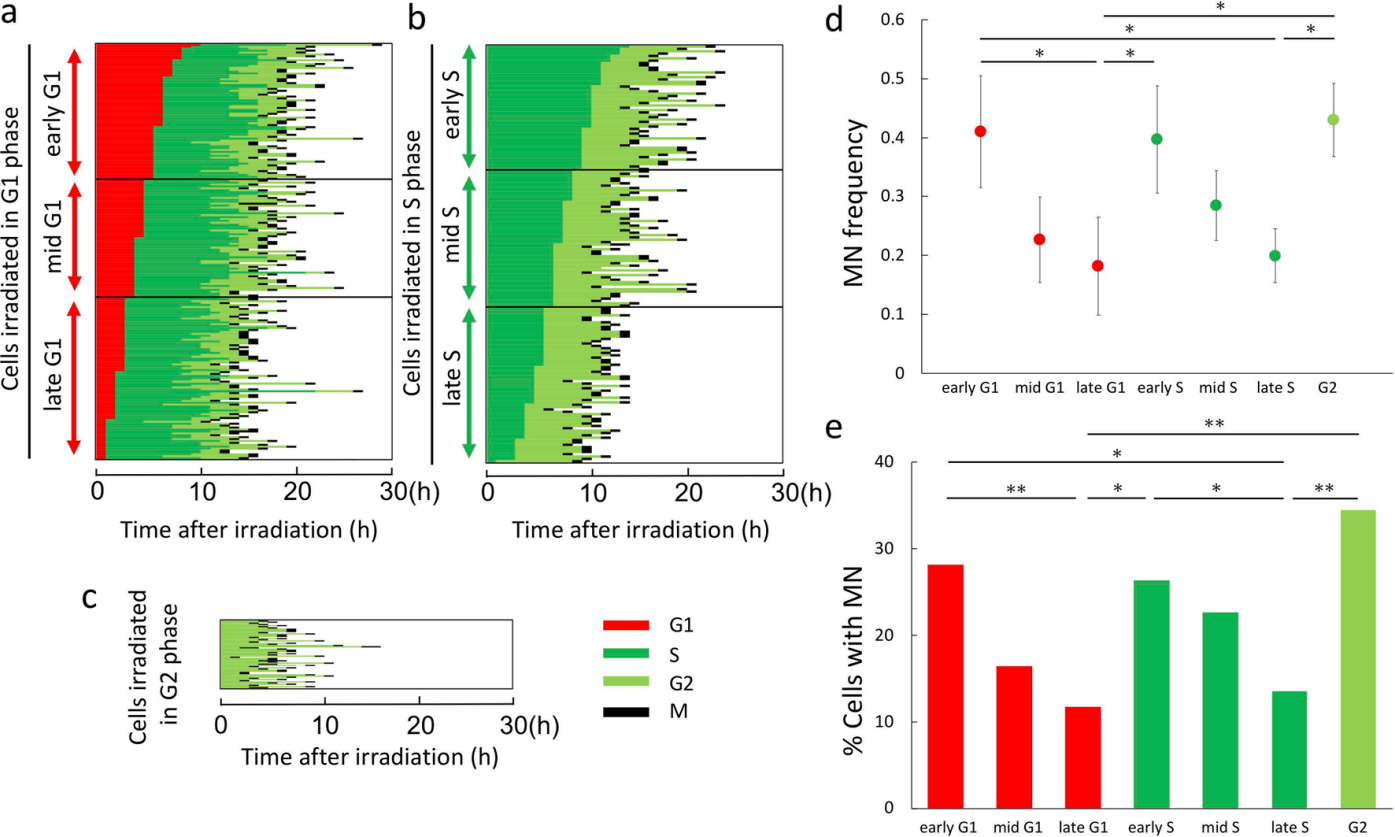
Sub-classification of cells in G1 and S phases at the time of irradiation based on the times at which they entered the next phase, and determination of their radioresponse. (**a**) Sub-classification of cells in G1 phase at the time of irradiation. Cells irradiated (2 Gy) in G1 phase were sorted according to their duration of G1 phase and sub-classified into early, mid-, and late sub-phases (each sub-phase group consisted of 61–85 cells). (**b**) Sub-classification of cells within S phase at irradiation. Cells irradiated (2 Gy) in S phase were sorted according to their durations of S phase and classified into early, mid-, and late sub-phases (each sub-phase group consisted of 53–66 cells). (**c**) Randomly sorted cells irradiated in G2 phase (cell number, 58). (**d**) MN frequency in cells irradiated in each sub-phase. Data are means ± SD of three independent experiments. ANOVA with post hoc Tukey’s multiple comparison test: *p < 0.05; **p < 0.01. (**e**) Proportions of cells with MNs in each sub-phase. Chi-square test: *p < 0.05; **p < 0.01.

HeLa-Fucci (CA)2 cells irradiated in G1 phase were confirmed not to undergo G1 arrest, as described above (Fig. 2a, upper panels). Therefore, on the basis of time-lapse imaging data, we assumed that cells with a shorter G1 phase were closer to S phase at the time of irradiation, whereas those with a longer G1 phase were closer to M phase. Along these lines, cells in G1 phase at irradiation (2 Gy) were sub-classified into early, mid-, and late phases, according to the lengths of their G1 phase (Fig. 4a). Because the extent of S-phase arrest in cells irradiated in S phase was quite small (Fig. 2b, upper panels), we applied the same approach to cells irradiated (2 Gy) in S phase, i.e., the cells were further sub-classified into early, mid-, and late phases (Fig. 4b). Due to the low efficiency of collection of G2 phase cells, we did not sub-classify cells irradiated (2 Gy) in G2 phase (Fig. 4c).

Next, we determined the MN frequencies for each cell, as shown in Fig. 4a–c. The mean values for each sub-phase are shown in Fig. 4d. As cells progressed from early G1 to late G1 or from early S to late S, they gradually exhibited reduced radioresponse scores, whereas after the cells entered G2 phase, they had higher scores. Notably, MN frequencies were significantly elevated between late G1 and early S phase. Similar results were also obtained when the proportion of cells with MNs was used as the endpoint (Fig. 4e), or when cells were irradiated with a higher dose (4 Gy) (Supplementary Fig. S1).

### Determination of sub-positions within G1 and S phases at the time of irradiation, according to the intensity of fluorescence and corresponding MN levels

**Figure 5:**
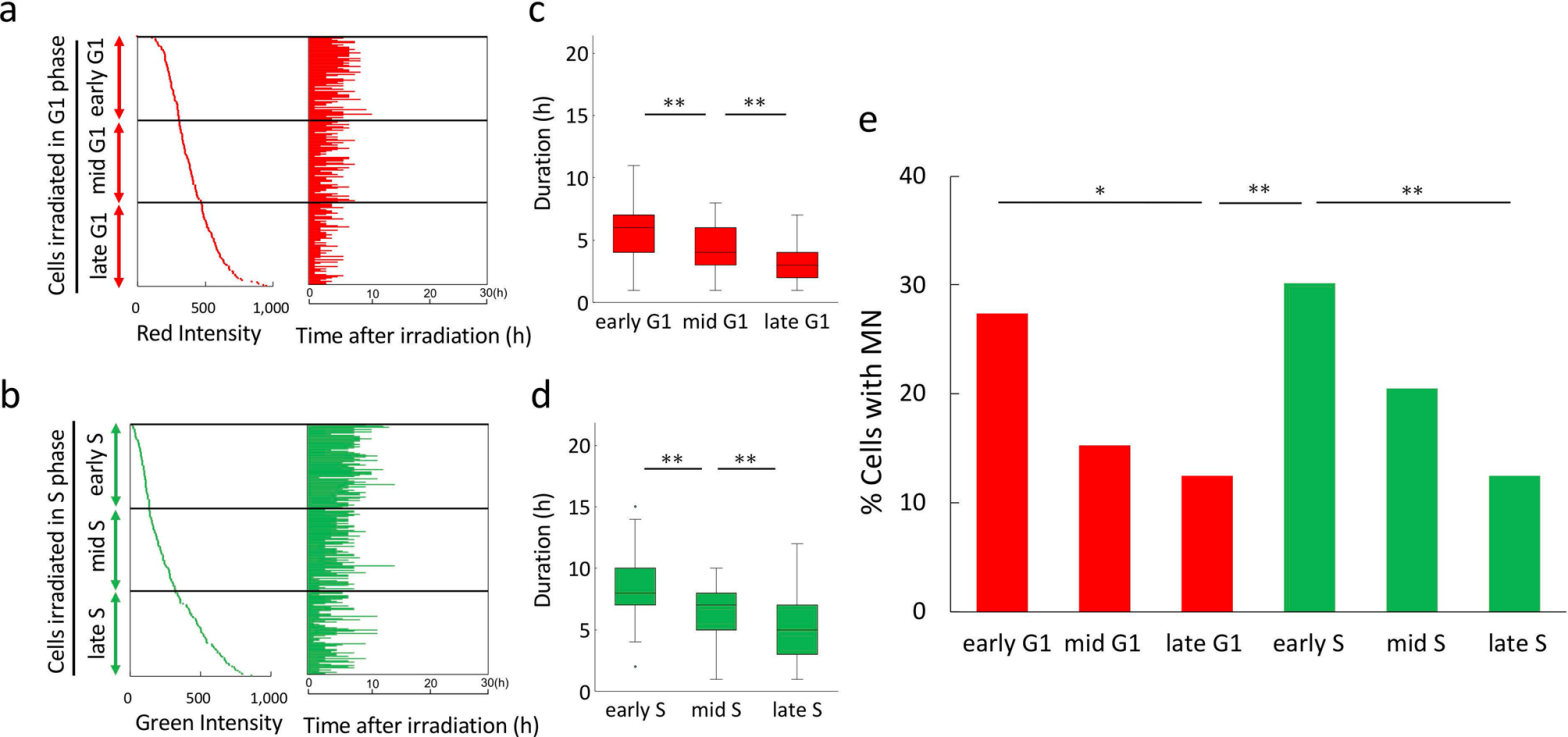
Sub-classification of cells within G1 and S phases at the time of irradiation based on their fluorescence intensity at irradiation, and determination of their radioresponse. (**a**) Sub-classification of cells in G1 phase according to their fluorescence intensity at the time of irradiation and their corresponding durations of G1 phase. Cells irradiated (2 Gy) in G1 phase were sorted according to their intensity of red fluorescence at the time of irradiation and classified into early, mid-, and late sub-phases (each sub-phase group consisted of 72 or 73 cells) (left panel). Each corresponding duration of G1 phase was extracted from time-lapse imaging data (right panel). (**b**) Sub-classification of cells within S phase according to their fluorescence intensity at irradiation, and the corresponding durations of S phase. Cells irradiated (2 Gy) in S phase were sorted according to their intensity of green fluorescence at irradiation and classified into early, mid-, and late sub-phases (each sub-phase group consisted of 72 or 73 cells) (left panel). Each corresponding duration of S phase was extracted from time-lapse imaging data (right panel). (**c**,**d**) Quantitative analysis of durations of each sub-G1 phase or sub-S phase at irradiation. Data are represented as box-whisker plots as described in Fig. 2. Mann–Whitney U-test: *p < 0.05; **p < 0.01. (**e**) Proportions of cells with MNs in each sub-phase. Chi‐square test: *p < 0.05; **p < 0.01.

To confirm these findings, we took an independent approach to determining the sub-positions within G1 and S phases at irradiation. As shown in a typical fluorescence image of HeLa-Fucci (CA)2 cells, various intensities of red and green fluorescence were observed (Supplementary Fig. S2a). Given that fluorescence intensities gradually increased as the cell cycle progressed during a given fluorescence phase (Supplementary Fig. S2b), we speculated that the intensities of red and green fluorescence at irradiation would reflect sub-positions during the G1 and S phases. Thus, cells within the G1 and S phases were sorted according to the intensity of fluorescence and classified into early, mid-, and late phases (Fig. 5a,b). The fluorescence intensity in each sub-group at irradiation and the corresponding times at which they entered the next cell cycle stage were correlated (Fig. 5c,d). The results confirmed that cells with higher fluorescence intensity tended to enter the next cell cycle phase sooner. We found that results related to radioresponse were essentially the same as those obtained using the previous approach (Fig. 5e).

### Confirmation of the results in HeLa-Fucci (SA) cells in G1 and early S phases

**Figure 6:**
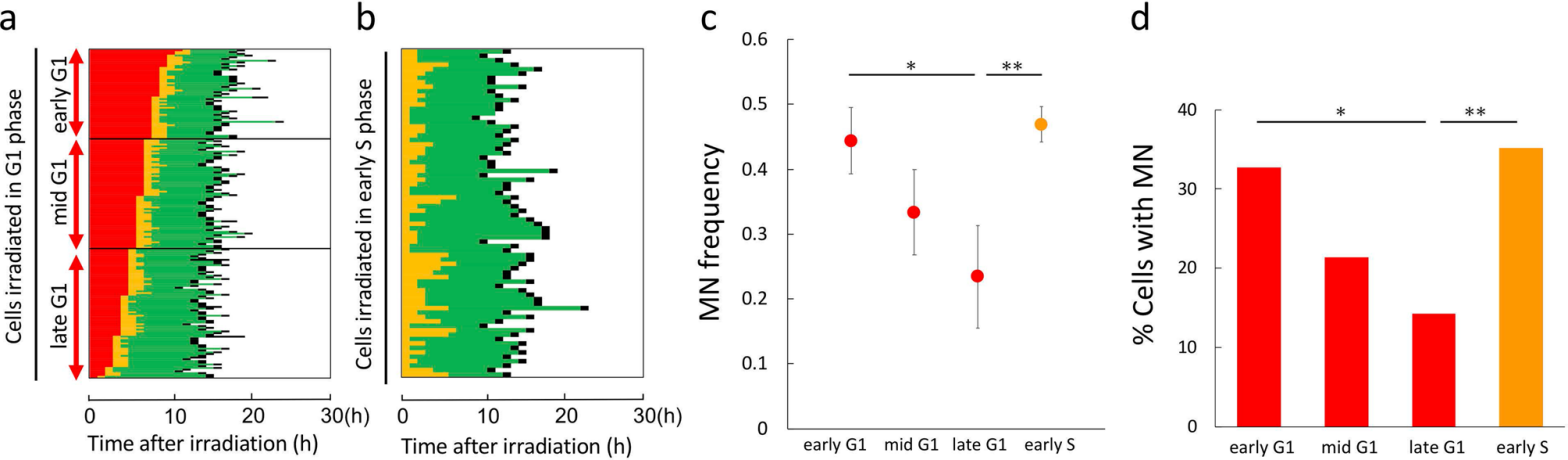
Sub-classification of HeLa-Fucci (SA) cells within G1 and S phases at irradiation based on the times at which they entered the next phase, and determination of their radioresponse. (**a**) Sub-classification of Fucci (SA) cells in G1 phase at the time of irradiation. Cells irradiated (2 Gy) in G1 phase were sorted according to their durations of G1 phase and classified into early, mid-, and late sub-phases (each sub-phase group consisted of 51–74 cells). (**b**) Randomly sorted cells irradiated in early S phase (cell number, 74). (**c**) MN frequency in cells irradiated in each sub-phase. Data are means ± SD of three independent experiments. ANOVA with post hoc Tukey’s multiple comparison test: *p < 0.05; **p < 0.01. (**d**) Proportions of cells with MNs in each sub-phase. Chi‐square test: *p < 0.05; **p < 0.01.

To further confirm the results described above, we used HeLa-Fucci (SA) cells. Although the S and G2 phases cannot be distinguished in this cell line, early S phase can be distinguished from other phases due to emission of both red and green fluorescence^8^. Cells irradiated in G1 phase were sorted according to the times at which they entered S phase, and sub-classified into early, mid-, and late phases (Fig. 6a). Cells irradiated in early S phase were extracted as shown in Fig. 6b. The results regarding radioresponse were essentially the same as those obtained in HeLa-Fucci (CA)2 cells (Fig. 6c,d).

## Discussion

In an effort to determine the accurate cell cycle-dependent fluctuation of radioresponse of HeLa cells, we used HeLa-Fucci (CA)2 cells, a cell cycle-visualisation system, in conjunction with the MN assay. This cell line was useful for validating issues related to using cell populations synchronised in M phase. We observed a rapid collapse of synchronicity even when we simulated a completely M phase-synchronised virtual cell population. By taking advantage of the properties of the system, we then further subdivided cells irradiated in G1 or S phase into early, mid-, and late sub-phases within each phase. Two independent approaches were taken, one based on the time at which the cell entered the next phase following irradiation in accordance with the time-lapse imaging data, and the other based on fluorescence intensity at the time of irradiation. Both results yielded essentially the same results, demonstrating that cells exhibited gradually reduced radioresponse as they progressed from early to late sub-phases during G1 and S phase. Cells in G2 phase exhibited a stronger radioresponse. The findings regarding early, mid-, and late G1 phases and early S phase were further confirmed using HeLa-Fucci (SA) cells.

Notably, our results obtained during G1 phase were a mirror image of those reported by Terasima and Tolmach, who reported that cells gradually become more radiosensitive as they approach the G1/S boundary^1,2^. Given the low sustainability of synchronicity, as shown in Fig. 1, a mixture of G1 and S phase cells are present around this time, and the greater number of cells in early S phase may result in a gradual increase in radiosensitivity, possibly masking the radioresistance of cells in late G1 phase. Thus, our approach could detect changes in radioresponse with higher resolution during the very short period between late G1 and early S phases. On the other hand, the pattern of changes during S phase was the same as in the previous report: cells had lower scores as they approached late S phase. The percentage of cells in S phase rose to ~ 100% around 12–16 h (Fig. 1), presumably representing a mixture of cells in mid- and late S phases. It is reasonable that increasing numbers of cells in late S phase resulted in a gradual increase in radioresistance around this stage. In addition, we showed that cells in G2 phase had higher scores. This was in conflict with the initial report by Terasima and Tolmach^1,2^, but we believed it was due to contamination with late S phase cells. Indeed, when cells in S phase were killed by incorporation of ^3^H-thymidine, the resultant pure population of G2 phase cells exhibited higher radiosensitivity^18^.

Previously, it was technically quite difficult to clearly differentiate between late G1 and early S phase (e.g., by DNA content); therefore, phenomena occurring around that stage have been considered together as the G1/S boundary. However, the Fucci system clearly distinguished between them, revealing that cells in each phase exhibit different radioresponses. Considering that solid tumour cells are thought to have a long G1 phase in vivo^19^, the fluctuation in radiosensitivity/radioresponse between late G1 and early S phase is highly expected. These findings have important clinical implications. Using the Fucci system as a cell cycle reporter, Otani et al. reported that the chemotherapeutic agent gemcitabine causes B16BL6 cells to arrest in early S phase, leading to enhancement of radiosensitisation in vitro and in vivo^20^. We previously showed using HeLa-Fucci (SA) cells that cells arrested at S phase, rather than G1, when exposed to low oxygen tension^21^. Interestingly, however, DNA content was quite close to that in G1 phase. It has been speculated that these cells could be in early to mid-S phase, as ribonucleotide reductase is inhibited under hypoxia and DNA synthesis is markedly suppressed^21^. Thus, tumour cells soon after reoxygenation from hypoxia following irradiation are likely to be radiosensitised by the next irradiation. Tumour cells with deficient p53 function do not exhibit G1 arrest, but instead undergo G2 arrest after the first irradiation, resulting in enhanced radiosensitisation when the next irradiation is given in a fractionated radiotherapy regimen. This is called “redistribution” and has been recognised as an important concept in fractionated radiotherapy^19^. If normal cells with functional p53 are irradiated, G1 arrest occurs at the G1/S boundary^19^. Using the Fucci system, we previously showed that cells arrested in red phase in human-TERT immortalised normal human diploid fibroblasts (BJ-hTERT)^10^. Taken together, these findings imply that in normal cells, arrest at this stage after the first irradiation may contribute to alleviation of damage from the next irradiation. It is well established that TGF-β induces G1 arrest by inducing CDK inhibitors near the G1/S boundary^22^, leading to the epithelial–mesenchymal transition (EMT)^23^. Sakaue-Sawano et al. clearly demonstrated that mouse NMuMG breast epithelial cells expressing Fucci (SA) accumulate in the red phase after TGF-β treatment^8^. Given that TGF-β induces radioresistance^24^, arrest in late G1 phase could be a mechanism of radioresistance in TGF-β-exposed tumour cells.

Hufnagl et al. proposed a model based on the local, sister chromatid conformation-dependent switch between non-homologous end joining (NHEJ) and homologous recombination (HR)^25^. Because the fidelity of HR is quite high, it was assumed that lethality of DSBs repaired by HR is much smaller than the lethality of DSBs repaired by NHEJ. Thus, cell survival is thought to increase as DNA replication progresses during S phase, reaching a peak at the late S phase. Their model successfully explained the fluctuation in radiosensitivity from G1 to G2 phases observed in Chinese hamster (V79) cells. However, cell lines with a substantially shorter G1 phase (e.g. V79), lack a higher radiosensitivity domain around the G1/S boundary^4–7^; such a domain is typically seen only in cell lines with a long G1 phase (e.g. HeLa), as described above^7^. Given that HR does not occur during G1 phase, their model did not explain radioresistance observed during G1 phase. The existence of other DSB repair pathways with fidelity comparable to HR that function only at the late G1 phase may explain the radioresistance; to date, however, only backup pathways have been reported so far and most of them are likely to be highly error-prone^26,27^. In another model, NHEJ initially attempts to repair DSBs, but if rapid rejoining does not take place, end resection occurs and could be replaced by HR^28–30^. HeLa cells in late G1 phase at the time of irradiation are expected to enter S phase soon thereafter due to the lack of G1 arrest, as shown in Fig. 2a, before they can be sufficiently repaired by NHEJ. The potential shift from NHEJ to end resection to HR may occur in cells irradiated at this stage. However, this does not explain the high radioresponse observed in early S phase. We speculate that collision of DSBs with initial events for DNA replication at this stage somehow interferes with the HR process. How the duration of G1 phase affects repair of DSBs produced during G1 phase, and exactly how radioresistance in late G1 phase and high radiosensitivity in early S phase are induced at the molecular level, remain intriguing issues to be addressed.

The present study has some limitations. The findings stem from only a single cell line of HeLa cells, therefore, further validation should be performed in other cell lines with a long G1 phase. Moreover, radiosensitivity was evaluated by an MN assay, but not a clonogenic assay. MNs frequently undergo nuclear envelope rupture, resulting in severe nuclear import defects^31^, and chromatin bridges can lead to chromothripsis^32^. Our method for detecting only MNs with nuclear envelope membranes likely underestimates the total abundance of micronuclei and cannot evaluate the cell’s proliferative capacity, which can be measured by a clonogenic assay. We cannot rule out the possibility that the discrepancy between this study and the previous is a result of this issue; however, many studies have shown a close correlation between conventional MN assays and clonogenic radiosensitivity^12–14^. To support our study, we tried to collect the two cell populations separately from the lowest and highest intensities of red fluorescence by FACS sorting of HeLa-Fucci(SA) cells; these cells correspond to early and late G1 phases, respectively, presumably not containing cells in S phase (Supplementary Fig. S3). Each cell population was then subjected to a colony assay. Our preliminary data showed that the surviving fractions following 6 Gy-irradiation were 0.057 ± 0.0070 and 0.076 ± 0.0056 (Student’s t test, p < 0.05, n = 3), respectively.

In this study, we for the first time revealed the unique pattern of radioresponse from G1 to G2 phase in HeLa cells, as determined by the MN assay. These findings may facilitate the development of strategies aimed at enhancing therapeutic gain; however, further validation is needed.

## Methods

### Cell lines and culture conditions

Two lines of HeLa cells expressing the Fucci probes HeLa-Fucci (SA) and HeLa-Fucci (CA)2 were provided by Riken BRC through the National Bio-Resource Project of MEXT, Japan. As described previously^9,10^, cells were maintained in DMEM (Sigma-Aldrich, St. Louis, MO, USA) containing a high concentration of glucose (4500 mg/L) with 100 units/mL penicillin and 100 μg/mL streptomycin, supplemented with 10% fetal bovine serum. Cells were cultured at 37 °C in a humidified 5% CO_2_ atmosphere.

### Irradiation

Cells were irradiated as described previously^33^, using a Clinac 6EX linear accelerator (Varian Medical Systems, Palo Alto, CA, USA) with a photon beam of nominal energy of 4 MV at a dose rate of 2.4 Gy/min. Calibration was performed at the isocenter underneath an acrylic plate of 5-cm thickness for a field size of 20 cm × 20 cm.

### Colony formation assay

Colony formation assay was performed as described previously^34^. To determine HeLa-Fucci (CA)2 radiosensitivity, an appropriate number of cells were seeded in 60-mm dishes in triplicate and immediately irradiated. After incubation for 10 days, the colonies were fixed using 4% paraformaldehyde, and then stained with crystal violet. Clonogenic survival was determined by counting colonies consisting of more than 50 cells. The surviving fractions were calculated from three independent experiments.

### Time-lapse imaging and pedigree analysis

Time-lapse imaging and pedigree analysis were performed as described previously^10^. Briefly, time-lapse images were acquired at 1-h intervals on a BIOREVO BZ-9000 fluorescence microscope (KEYENCE, Osaka, Japan). During imaging, cells were maintained in an incubation chamber at 37 °C in a humidified atmosphere containing 95% air/5% CO_2_ (Tokai Hit, Fujinomiya, Japan). Each cell was monitored for 48 h after irradiation, and changes in fluorescence colours and their durations were recorded. Pedigree analysis was performed using time-lapse imaging data until the next mitosis. Intensity of Fucci fluorescence was calculated by subtracting red and green background fluorescence intensity from the mean fluorescence intensity of Fucci red (mCherry) and green (mVenus) fluorescence within the nucleus, respectively, using software equipped in the fluorescence microscope.

### Determination of sub-positions within G1 and S phases

On the basis of pedigrees acquired from time-lapse imaging data, cells were sorted in descending order according to the lengths of their G1 or S phase. Each sorted cell population was first divided so that the early, mid, and late sub-phase groups contained the same number of cells, and then cells with the same time lengths that straddled these sub-phases were assigned to one of the phases so that they belonged to a single group. For the fluorescence intensity-based classification, cells were sorted in ascending order of the intensities of red and green fluorescence for cells irradiated in G1 and S phases, respectively. Each sorted cell population was divided so that the early, mid, and late sub-phase groups contained the same cell number of cells.

### Micronucleus assay

The micronucleus (MN) assay was used to evaluate radioresponse of cells irradiated at various cell cycle phases. Using time-lapse imaging data, micronuclei were counted in two daughter cells in G1 phase after mitosis. Radioresponse was expressed as mean number of MNs per paired-daughter cell after mitosis (MN frequency) or the proportion of paired-daughter cells that contained MNs in all counted pairs.

### Cell sorting

To separately collect HeLa-Fucci (SA) cells in early and late G1 sub-phases, cells with lower and higher red fluorescence intensity were sorted according to Fucci fluorescence intensity as described previously^35^. Fluorescence analysis and cell sorting were carried out using a MoFlo XDP flow cytometer (Beckman Coulter, Brea, CA, USA). After sorting, cells were subjected to colony‐forming assays as described above.

### Statistical analysis

Statistical analyses were performed as described previously^33,34^. Mann–Whitney *U* test, one-way ANOVA with post hoc Tukey's multiple comparison test, Student’s t-test, or chi‐square test was used as appropriate. P-values < 0.05 were considered statistically significant.

## Supplementary information

Supplementary Information.
