# Supplementary material for: Fluctuation in radioresponse of HeLa cells during the cell cycle evaluated based on micronucleus frequency
Source: Sci Rep. 2020 Nov 30;10:20873. doi: 10.1038/s41598-020-77969-0 (PMC7705701; doi:10.1038/s41598-020-77969-0)
Supplement: Supplementary file 1 — Supplementary Information. [file 41598_2020_77969_MOESM1_ESM.pptx]

## Slide 1
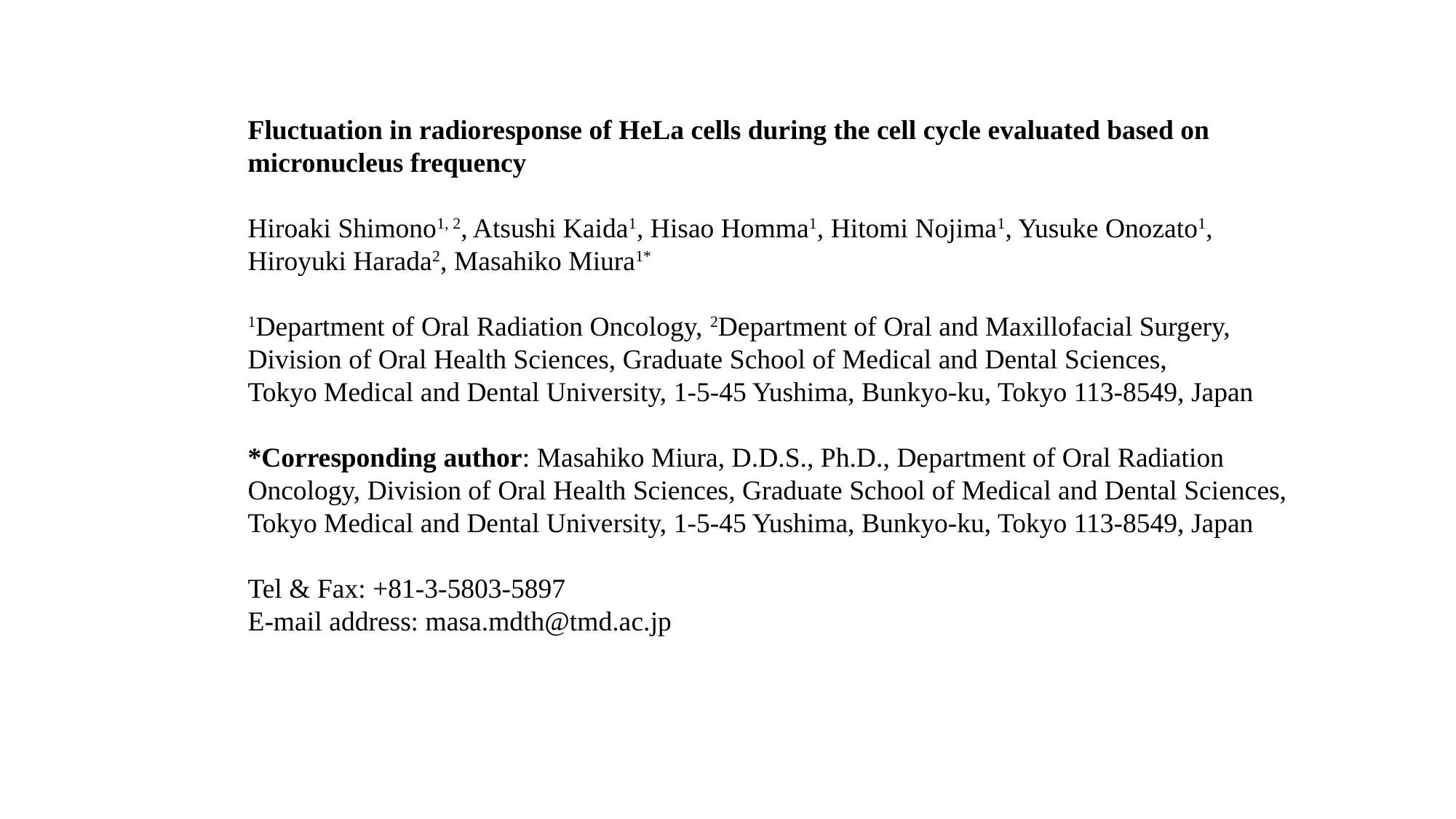

Fluctuation in radioresponse of HeLa cells during the cell cycle evaluated based on
micronucleus frequency
Hiroaki Shimono1, 2, Atsushi Kaida1, Hisao Homma1, Hitomi Nojima1, Yusuke Onozato1,
Hiroyuki Harada2, Masahiko Miura1*
1Department of Oral Radiation Oncology, 2Department of Oral and Maxillofacial Surgery,
Division of Oral Health Sciences, Graduate School of Medical and Dental Sciences,
Tokyo Medical and Dental University, 1-5-45 Yushima, Bunkyo-ku, Tokyo 113-8549, Japan
*Corresponding author: Masahiko Miura, D.D.S., Ph.D., Department of Oral Radiation
Oncology, Division of Oral Health Sciences, Graduate School of Medical and Dental Sciences,
Tokyo Medical and Dental University, 1-5-45 Yushima, Bunkyo-ku, Tokyo 113-8549, Japan
Tel & Fax: +81-3-5803-5897
E-mail address: masa.mdth@tmd.ac.jp

## Slide 2
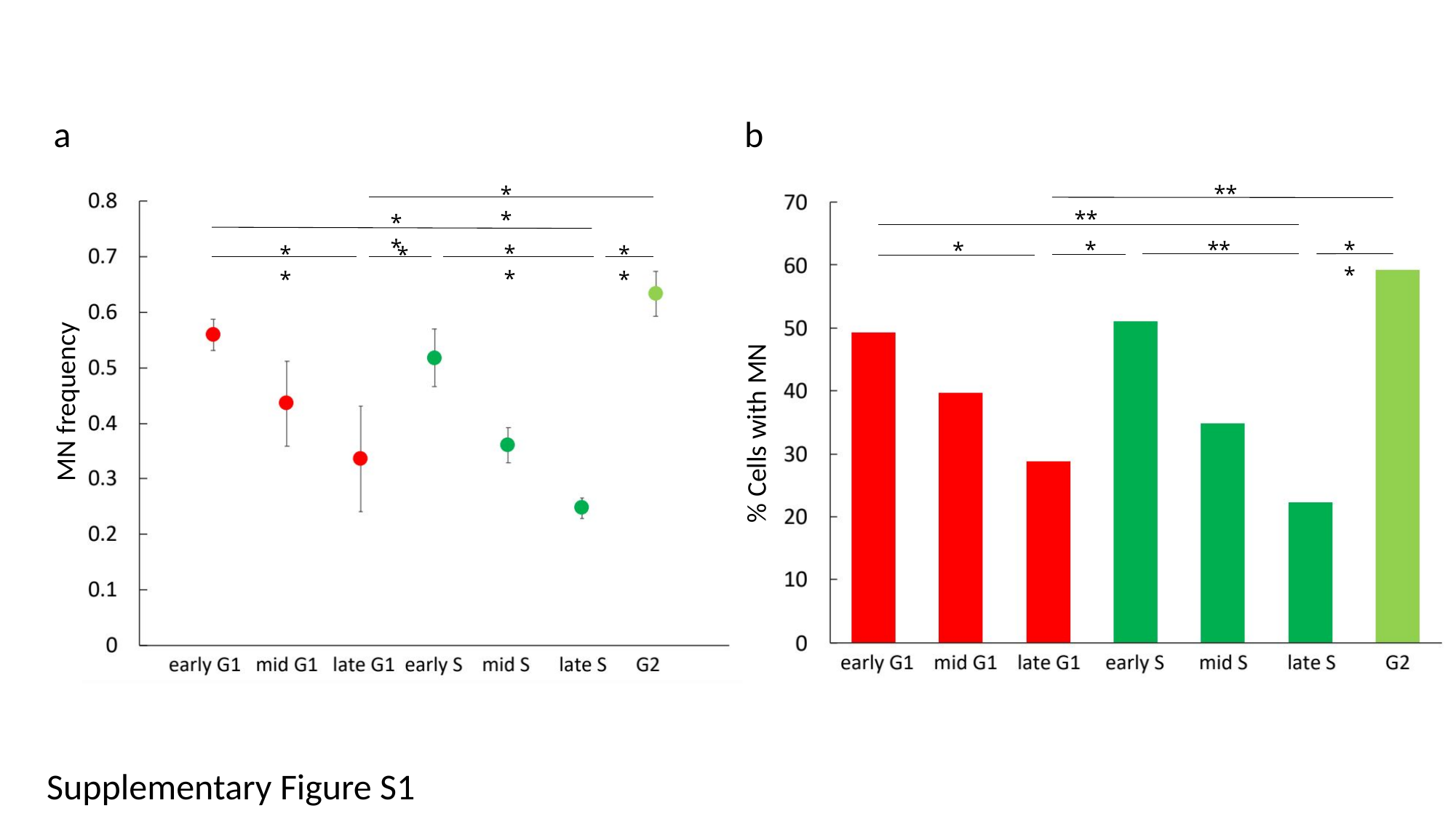

a
b
**
**
**
**
**
*
**
*
**
**
**
*
MN frequency
% Cells with MN
Supplementary Figure S1

## Slide 3
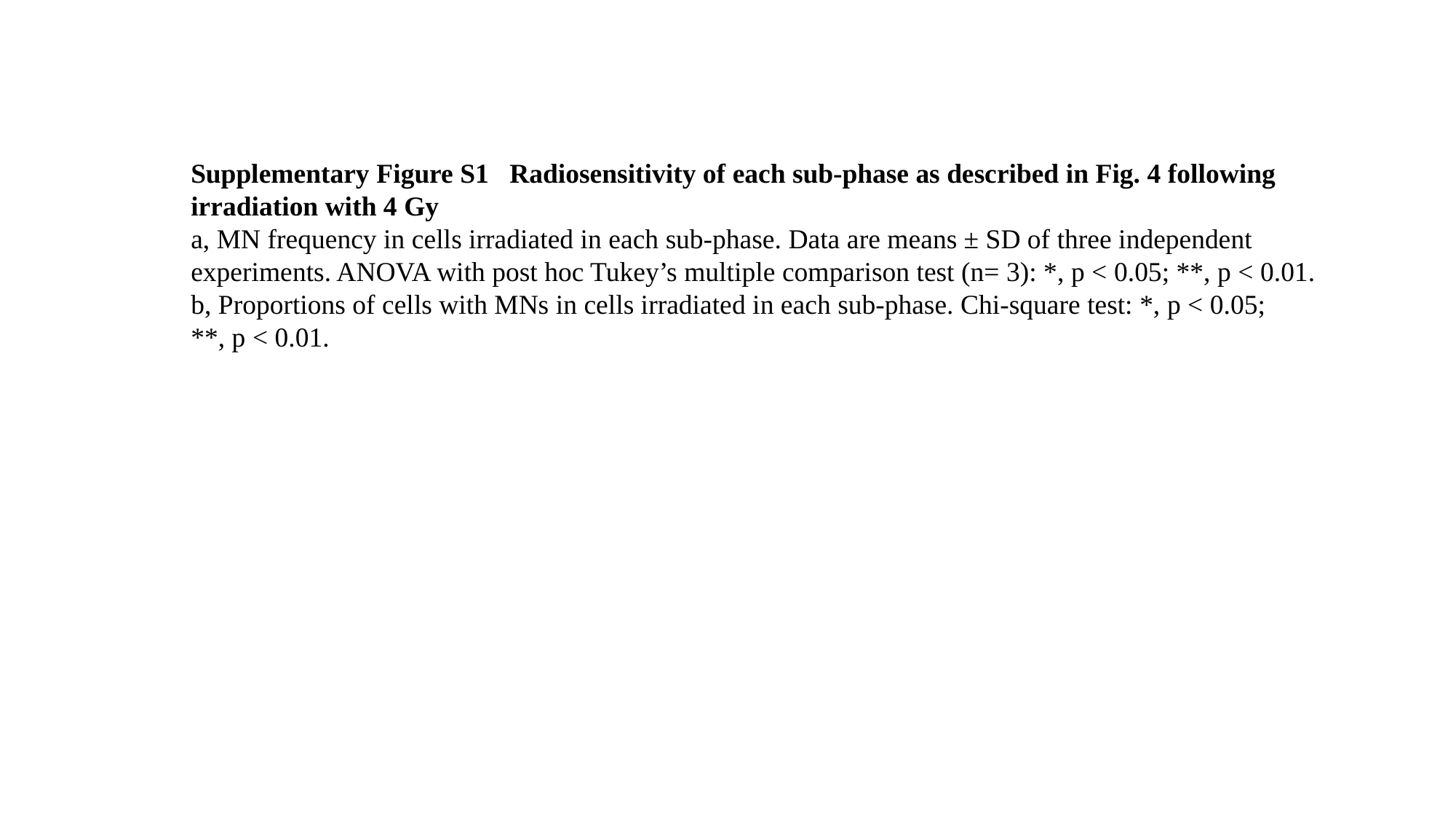

Supplementary Figure S1 Radiosensitivity of each sub-phase as described in Fig. 4 following
irradiation with 4 Gy
a, MN frequency in cells irradiated in each sub-phase. Data are means ± SD of three independent
experiments. ANOVA with post hoc Tukey’s multiple comparison test (n= 3): *, p < 0.05; **, p < 0.01.
b, Proportions of cells with MNs in cells irradiated in each sub-phase. Chi-square test: *, p < 0.05;
**, p < 0.01.

## Slide 4
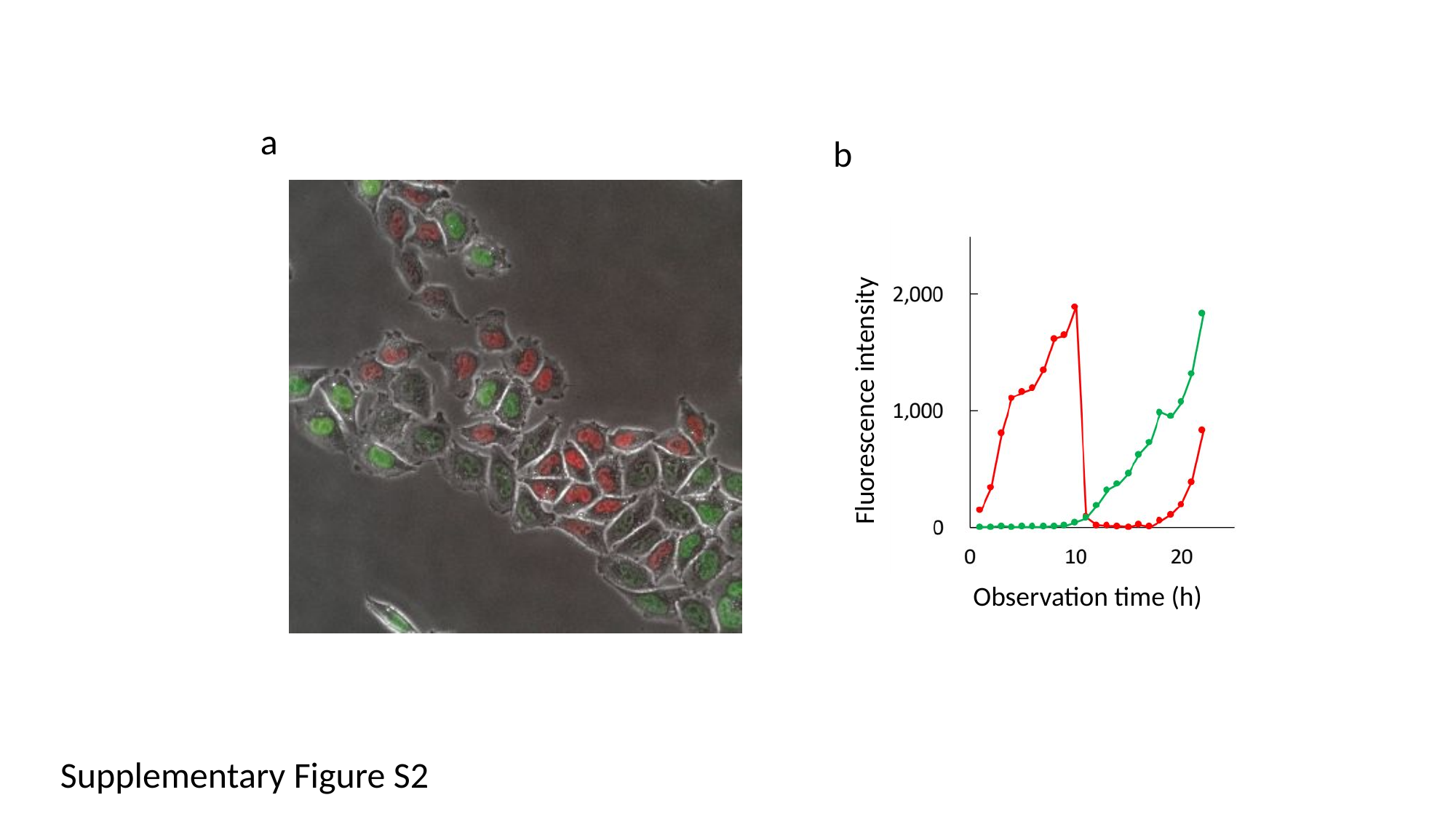

a
b
Fluorescence intensity
Observation time (h)
Supplementary Figure S2

## Slide 5
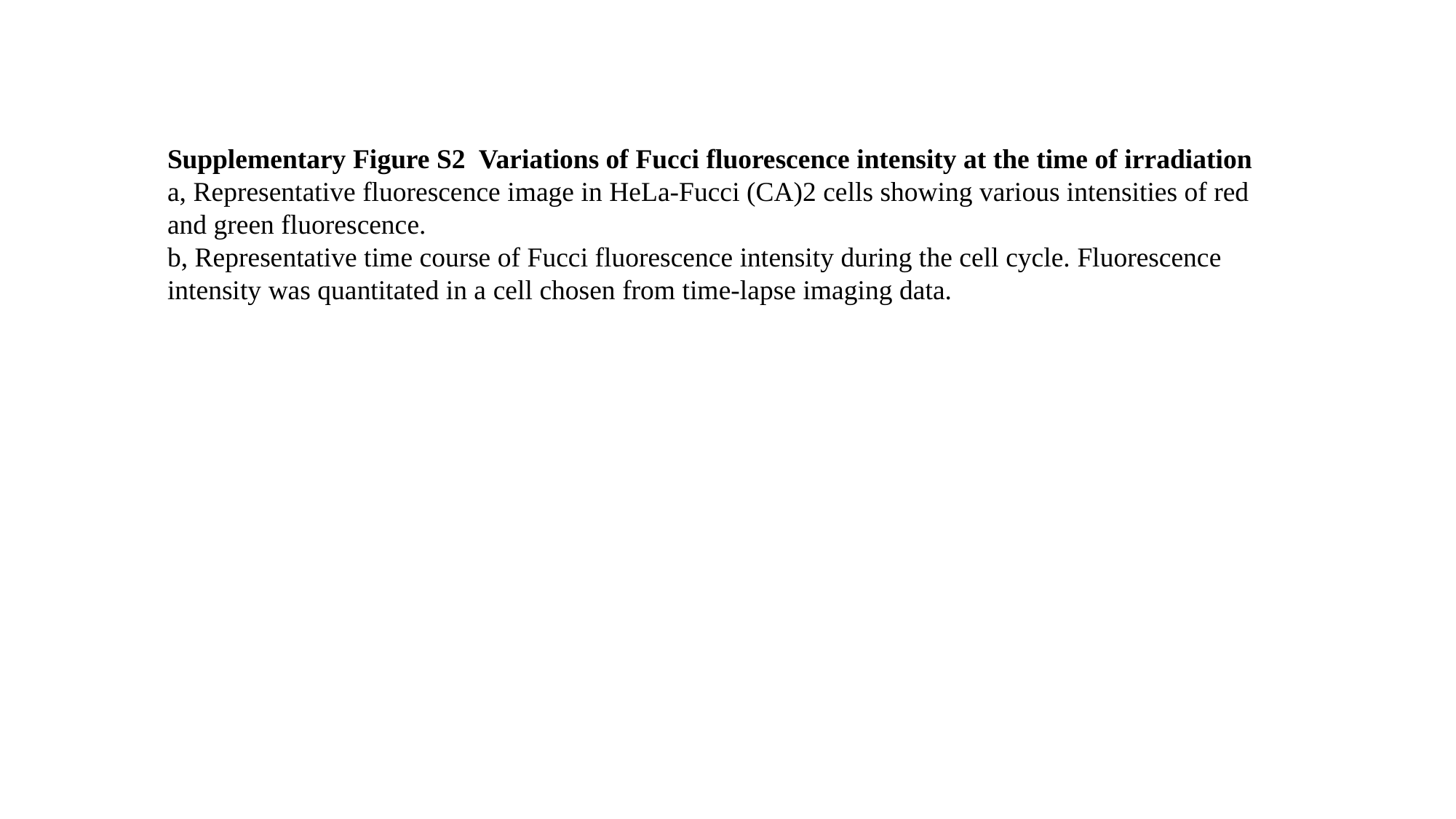

Supplementary Figure S2 Variations of Fucci fluorescence intensity at the time of irradiation
a, Representative fluorescence image in HeLa-Fucci (CA)2 cells showing various intensities of red
and green fluorescence.
b, Representative time course of Fucci fluorescence intensity during the cell cycle. Fluorescence
intensity was quantitated in a cell chosen from time-lapse imaging data.

## Slide 6
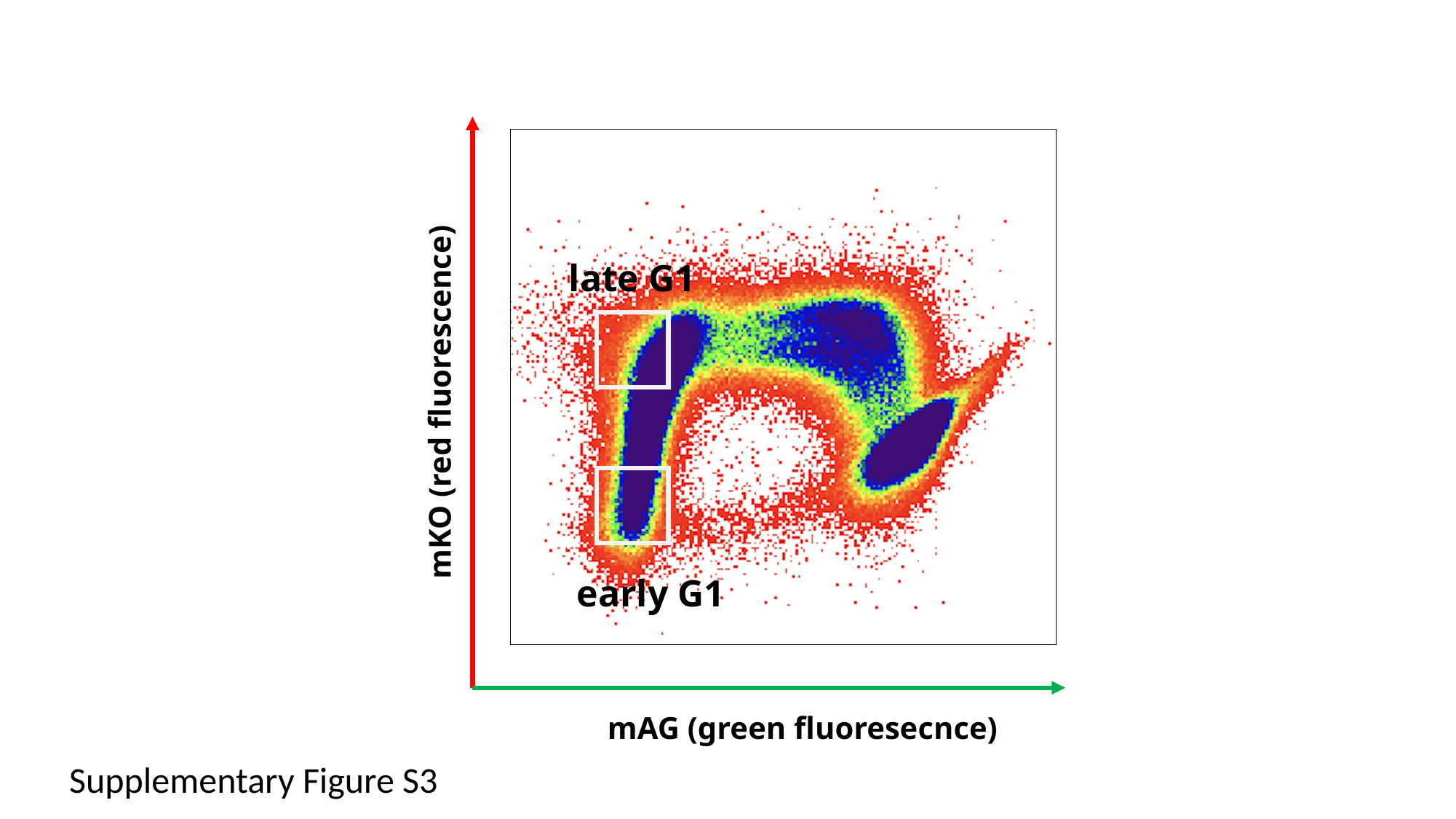

late G1
mKO (red fluorescence)
early G1
mAG (green fluoresecnce)
Supplementary Figure S3

## Slide 7
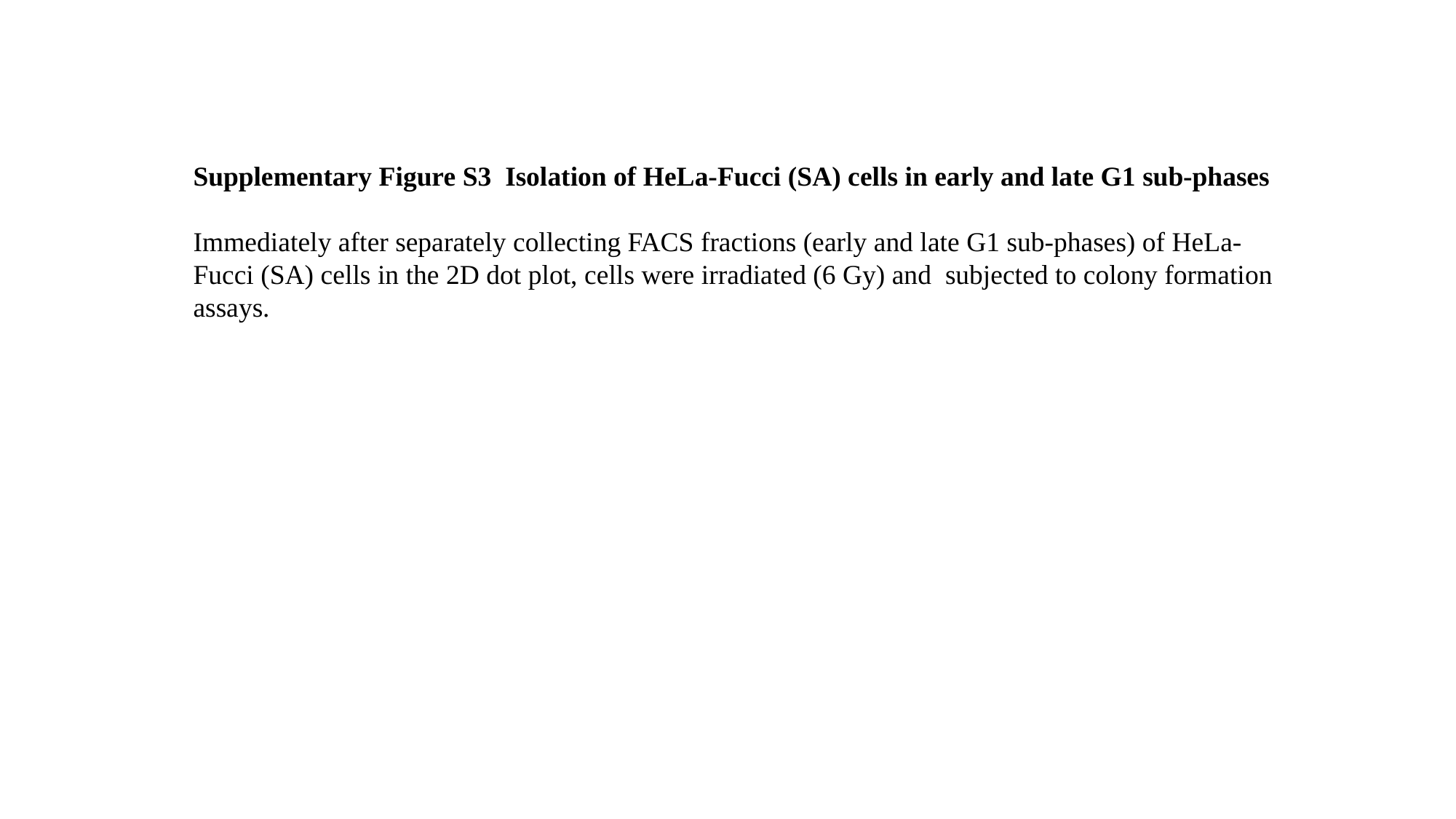

Supplementary Figure S3 Isolation of HeLa-Fucci (SA) cells in early and late G1 sub-phases
Immediately after separately collecting FACS fractions (early and late G1 sub-phases) of HeLa-Fucci (SA) cells in the 2D dot plot, cells were irradiated (6 Gy) and subjected to colony formation assays.
